# Dermatofibrosarcoma Protuberans in a Child: A Case Report

**DOI:** 10.1155/2012/796818

**Published:** 2012-12-18

**Authors:** Prashant Goyal, Shelly Sehgal, Sompal Singh, Shalabh Rastogi

**Affiliations:** ^1^Department of Pathology, Swami Dayanand Hospital, Shahdara, New Delhi 110095, India; ^2^Department of Otorhinolaryngology, Swami Dayanand Hospital, Shahdara, New Delhi 110095, India

## Abstract

*Background*. Dermatofibrosarcoma protuberans (DFSP) is an intermediate grade soft tissue neoplasm originating from the dermal layer of the skin. It usually occurs in adults; however, it can rarely be seen in infancy and childhood. Diagnosis of DFSP in children is quite difficult-given-rarity of this lesion, its variegated appearance, and its presentation sometimes at unusual sites. *Case*. We present the case of five-year-old boy who came with painless lesion on a forehead. Fine needle aspiration cytology (FNAC) suggested possibility of mesenchymal neoplasm. Patient was advised excision biopsy. Final diagnosis of DFSP was made based on histopathological findings. The patient was then advised reexcision surgery with wide margins. The patient was lost to followup and later turned up after two months with recurrence of a similar swelling at the same site. *Conclusion*. DFSP in children is rare and difficult to diagnose. Treatment of childhood DFSP is often delayed leading to incomplete excision. Hence, there is need to recognize and appropriately manage this uncommon childhood neoplasm.

## 1. Introduction

Dermatofibrosarcoma protuberans (DFSP) is a rare, slow-growing, intermediate grade neoplasm originating from the dermal layer of the skin [1]. It is most often found on the trunk and proximal limbs and is rare on the head and neck. It commonly favors young to middle aged adults. However, it can also occur in infancy and childhood. Because of the rarity of the tumor and its variegated appearance, diagnosis of DFSP in children is quite difficult. We are presenting this case to document the uncommon clinical presentation of DFSP in pediatric age group.

**Key Message**

DFSP in children is a rare and difficult diagnosis particularly when presenting at uncommon site like head and neck. Treatment of childhood DFSP is often delayed because of misdiagnosis leading to local growth and incomplete excision. Hence, pediatricians should be aware of this uncommon entity and should ensure wide excision and reduce risk of recurrence.

## 2. Case Report

**Figure 1:**
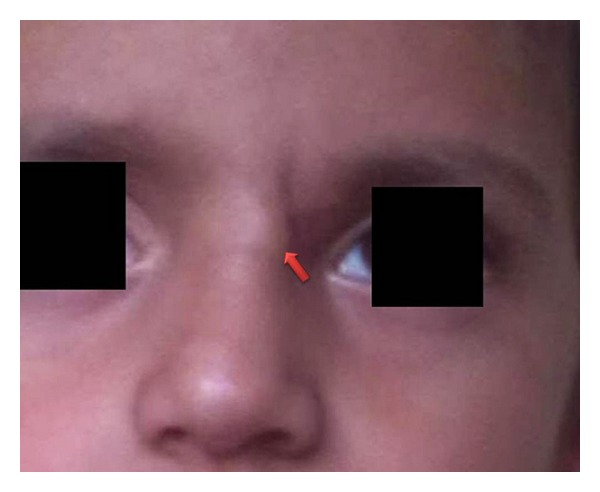
Photograph of patient showing slightly raised lesion on the forehead near the medial side of the left eyebrow.

We present a case of a five-year-old boy who presented with a slightly raised, painless lesion on base of the nose near the medial side of a the left eyebrow (Figure 1) since 6 months. On examination, there was nontender, firm nodule, measuring 0.5 cm in diameter. A fine needle aspiration cytology (FNAC) was performed with 23 G needle, and provisional diagnosis of mesenchymal neoplasm was rendered. The patient was advised excision biopsy for final diagnosis and behavior of neoplasm. Subsequently an excision biopsy was performed, and final diagnosis of DFSP was made based on the histopathological findings. 

The patient was then advised reexcision surgery with wide margins. Patient was lost to followup and later turned up after two months with recurrence of similar swelling at same site.

**Figure 2:**
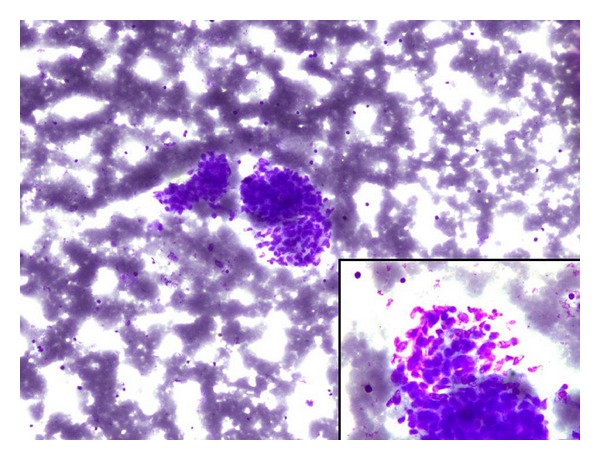
Cytosmear showing loosely cohesive clusters comprising oval to spindle cells in hemorrhagic background (10x, MGG) and inset showing tumor cells having oval hyperchromatic nuclei, inconspicuous nucleoli, and poorly defined cytoplasmic borders (40x, MGG).

**Figure 3:**
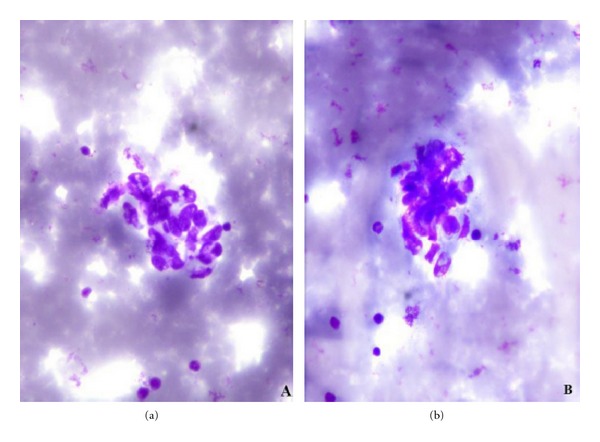
Cytosmear showing loosely cohesive clusters comprising cells with oval to spindle nuclei ((a) and (b)) (40x, MGG).

Pathological Finding: May-Grünwald Giemsa (MGG) stained cytological smears showed scanty cellularity comprising few cohesive clusters and singly dispersed oval to spindle cells in hemorrhagic background (Figures 2 and 3). Tumor cells had oval hyperchromatic nuclei, inconspicuous nucleoli, and poorly defined cytoplasmic borders (Figure 2 inset). Provisional diagnosis of mesenchymal neoplasm was rendered. The patient was advised excision biopsy. 

**Figure 4:**
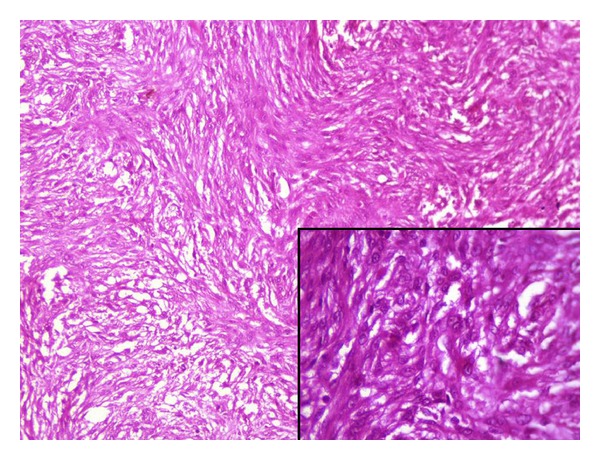
Section showing spindle cells arranged in short fascicles and storiform pattern (10x, H&E) and inset showing tumor cells with oval nuclei, vesicular chromatin, inconspicuous nucleoli, and scanty to moderate cytoplasm (40x, H&E).

**Figure 5:**
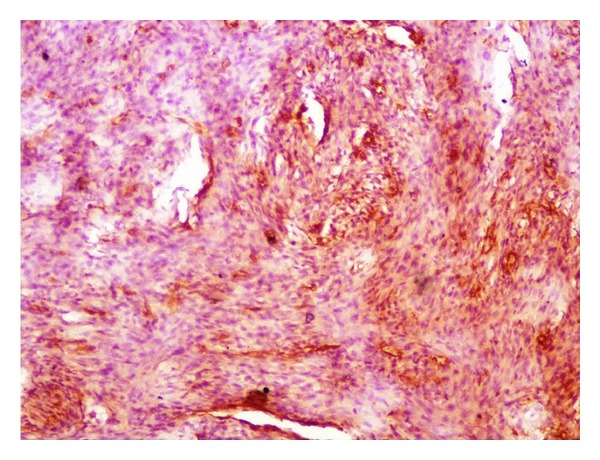
Section showing tumor cells focally positive for CD34 (10x, CD34 DAB).

Subsequently an excision biopsy was performed. We received tiny skin covered soft tissue, measuring 0.8 × 0.6 × 0.4 cm. Hematoxylin and eosin stained sections showed cellular and poorly circumscribed tumor in the dermis comprising interwoven bundles and fascicles of uniform spindle shaped cells arranged in “storiform” or “cartwheel” pattern (Figure 4). The tumor cells had monotonous appearance with oval nuclei, vesicular chromatin, inconspicuous nucleoli, and scanty to moderate cytoplasm (Figure 4 inset). The mitotic activity was moderate (3–5/10HPF). The tumor was seen infiltrating into the underlying subcutis. Diagnosis of DFSP was made. A panel of immunohistochemistry (IHC) comprising vimentin and CD-34 was applied. Tumor cells were positive for both vimentin and CD-34 (Figure 5). 

## 3. Discussion

DFSP is a slow-growing, locally aggressive tumor of intermediate malignancy with a marked tendency to local recurrence, but which rarely metastasizes [1]. Although historically it has been attributed to fibroblastic origin, recent IHC evidence suggests that it may arise from the dendritic cell in the skin. In 1924, Darier and Ferrand first described the entity of DFSP as a “progressive and recurring dermatofibroma” underscoring its predilection for local recurrence. Hoffman [2] reported three new cases and proposed the term DFSP in 1925. This tumor is uncommon usually present between 20 and 50 years of age, and is rare in children aged less than 16 years. It is most often found on the trunk and proximal limbs and is rare on the head and neck [3]. Trunk and limbs are also most common sites in children [4, 5]. It usually presents as a violaceous or bluish erythematous plaque or atrophic plaque or macule that is vascular in appearance. The size of tumor is varying from a few millimeters to a few centimeters [6, 7]. Clinically it is also confused with other lesions such as sclerosing basal cell carcinoma, morphea, scar, and anetoderma. Clinical diagnosis of DFSP in infancy and childhood may be awkward because, in the early stages, the tumor often looks like a vascular malformation [7]. 

Cytological smears of DFSP usually show scanty to moderate cellularity comprising scattered single cells and clusters with a storiform pattern and naked nuclei. The oval to spindle nuclei contain fine homogeneous chromatin with small nucleoli [8]. Similar findings were seen in our aspirate smears also. Histopathological examination reveals a highly cellular neoplasm consisting of spindle cells arranged primarily in storiform pattern. Subcutaneous involvement is characterized by growth within septa and between adipocytes. Our histomorphology also showed typical features, characteristic of DFSP. IHC studies have shown that the tumor cells in DFSP contain vimentin and actin (focally and inconstantly) and CD34 (strongly and consistently), but they are negative for S100 protein, HMB-45, keratin, and FXIIIa [9].

The DFSP in pediatric age group should be differentiated with other fibrohistiocytic tumors like dermatofibroma, leiomyoma, neurofibroma, dermatomyofibroma, infantile myofibromatosis, and fibrous hamartoma of infancy [3]. Dermatofibroma is a more nodular lesion that is limited to the dermis. Unlike DFSP, an IHC study shows that it is CD34 negative and factor XIIIa positive. Leiomyoma is composed of spindle cells with cigar-shaped nuclei showing positivity for desmin and caldesmon. Neurofibroma has smaller, wavy nuclei and is positive for S-100. Dermatomyofibroma is composed of bundles of monomorphic spindle cells arranged parallel to the epidermis expressing smooth-muscle actin and vimentin. Infantile myofibromatosis is characterized by spindle cells arranged in short interlaced bundles. The cellular aggregates are separated by fine bands of collagen, and vascular spaces can often be observed in the center of the tumor. Fibrous hamartoma of infancy is characteristic organoid pattern that is made up of fibrous collagenous trabeculae, primitive mesenchymal cells, and mature adipose tissue.

 DFSP in children is often misdiagnosed, leading to delay in treatment. Wide local excision (WLE) is necessary, since DFSP often exhibits extensive infiltration beyond gross margins. Particularly for neoplasm developing in the head and neck area it has been shown that they have a high rate of recurrence even after wide local excision with 2- to 3-cm margins. In order to achieve negative resection margins and simultaneously preserve the uninvolved tissue from resection, some authors suggest the use of Moh's micrographic surgery [10]. Adjuvant radiotherapy reduces the local recurrence rate when wide local excision is impossible because of anatomical constraints or functional and cosmetic concerns. Preoperative MRI may be used in children to delineate the size and extent of the lesion prior to surgery [1]. Imatinib mesylate, a selective tyrosine kinase inhibitor, is indicated in patients with unresectable, locally advanced, recurrent or metastatic disease [10].

## 4. Conclusion

DFSP in children is a rare and difficult to diagnose particularly when presenting at uncommon site like head and neck. Treatment of childhood DFSP is often delayed because of misdiagnosis leading to local growth and incomplete excision. Hence, pediatricians should be aware of this uncommon entity and should ensure wide excision and reduce risk of recurrence. 

## References

[1] Weedon D (2010). Tumors and tumors-like proliferations of fibrous and related tissues. *Weedon's Skin Pathology*.

[2] Hoffman E (1925). Uber das knollentreibende fibrosarkom de haut. *Dermatol Z*.

[3] Bhambri S, Desai A, Del Rosso JQ, Mobini N (2008). Dermatofibrosarcoma protuberans: a case report and review of the literature. *Journal of Clinical and Aesthetic Dermatology*.

[4] Iqbal CW, Peter S, Ishitani MB (2011). Pediatric dermatofibrosarcoma protuberans: multi-institutional outcomes. *Journal of Surgical Research*.

[5] Zaraa I, Ben-abdallah M, Driss M (2011). Dermatofibrosarcoma protuberans in children. *Archives de Pediatrie*.

[6] Sathyanarayana BD (2004). Childhood onset dermatofibrosarcoma protuberans. *Indian Journal of Dermatology, Venereology and Leprology*.

[7] Weinstein JM, Drolet BA, Esterly NB (2003). Congenital dermatofibrosarcoma protuberans: variability in presentation. *Archives of Dermatology*.

[8] Domanski HA (2005). FNA diagnosis of dermatofibrosarcoma protuberans. *Diagnostic Cytopathology*.

[9] Rosai J (2004). Skin: tumors and tumorlike conditions. *Rosai and Ackerman's Surgical Pathology*.

[10] Angouridakis N, Kafas P, Jerjes W (2011). Dermatofibrosarcoma protuberans with fibrosarcomatous transformation of the head and neck. *Head and Neck Oncology*.

